# Development of a Risk Predictive Model for Erectile Dysfunction at 12 Months after COVID-19 Recovery: A Prospective Observational Study

**DOI:** 10.3390/jcm13195757

**Published:** 2024-09-27

**Authors:** Fernando Natal Alvarez, Maria Consuelo Conde Redondo, Nicolas Sierrasesumaga Martin, Alejandro Garcia Viña, Carmen Marfil Peña, Alfonso Bahillo Martinez, Mario Jojoa, Eduardo Tamayo Gomez

**Affiliations:** 1 Department of Urology, Clinic University Hospital of Valladolid, 47003 Valladolid, Spain; 2 Urología Clínica Bilbao, Clínica IMQ Zorrotzaurre, 48014 Bilbao, Spain; 3 Department of Urology, Río Hortega University Hospital, 47003 Valladolid, Spain; 4 Department of Urology, Santa Barbara Hospital of Soria, 42005 Soria, Spain; 5 University of Valladolid, 47002 Valladolid, Spain; 6 BioCritic, Group for Biomedical Research in Critical Care Medicine, 47003 Valladolid, Spain; 7 Centro de Investigación Biomédica en Red de Enfermedades Infecciosas (CIBERINFEC), Instituto de Salud Carlos III, 28029 Madrid, Spain; 8 Anesthesiology and Critical Care, Clinic University Hospital of Valladolid, 47003 Valladolid, Spain; 9 Department of Surgery, Faculty of Medicine, University of Valladolid, 47005 Valladolid, Spain

**Keywords:** COVID-19, erectile dysfunction, cardiovascular disease, post-acute COVID-19 syndrome, logistic models

## Abstract

**Objectives**: To develop a risk prediction model for the identification of features involved in the prediction of erectile dysfunction (ED) at 12 months following COVID-19 recovery. **Methods**: We performed an observational prospective multicentre study. Participants were classified according to their history of COVID-19: (I) patients with a past history of COVID-19 and (II) patients without a previous microbiological diagnosis of COVID-19. A total of 361 patients (past history of COVID-19, n = 166; no past history of COVID-19, n = 195) were assessed from January 2022 to March 2023. Patients with a past history of COVID-19 were assessed at 12 months following COVID-19 recovery. The primary outcome measure was ED, assessed through the 5-item International Index of Erectile Function (IIEF-5). Data concerning epidemiologic variables, comorbidities and active treatment were also collected. We performed a binary logistic regression to develop a risk predictive model. Among the models developed, we selected the one with the higher Area Under the Curve (AUC). **Results**: The median age was 55 years in both groups. The ED prevalence was 55.9% in patients with past history of COVID-19 and 44.1% in those with no past history of COVID-19. The best predictive model developed for ED comprised 40 variables and had an AUC of 0.8. **Conclusions**: We developed a regression model for the prediction of ED 12 months after COVID-19 recovery. The application of our predictive tool in a community setting could eventually prevent the adverse effects of ED on cardiovascular health and the associated unfavourable economic impact.

## 1. Introduction

Erectile dysfunction (ED) is the inability to attain or maintain an erection sufficient for performing sexual intercourse [1]. The prevalence worldwide is variable between the different geographic regions, but is high and growing worldwide, reaching 42% in the US and 42.6–48.6% in Europe [1]. A previous study estimated that the worldwide population affected by ED may reach 322 million people by 2025, meaning an increase of 115% since 1995 [2]. Up to 50% of patients do not seek treatment, resulting in the infradiagnosis of the disease [3]. Apart from the medical aspect, this disease has an economic impact, with a loss of productivity estimated to be $7270 per worker per year in the US [4].

Several diseases have been related to ED, with COVID-19 being one of the most recent. Since its description [5], COVID-19 has been known to be an infectious disease that has led to a great variety of complications [6,7,8] in affected individuals, with a strong socioeconomic impact [9]. Some of them will last for a long time, which has led to the description of a condition known as long COVID [10]. SARS-CoV-2 vaccination strategies have been implemented to try to change the natural history of the disease, but a number of serious adverse effects have been described [11]. Since Sansone et al. described a relationship between COVID-19 and ED for the first time [12], other investigations [13,14] reported similar findings. Concerning the duration of ED following COVID-19, a study by Gök et al. showed that ED seems to last for at least one year after COVID-19 recovery [15].

Nevertheless, to date, no predictive tool has been created to assess the risk of developing post-COVID-19 ED. COVID-19 is a disease with a complex pathogenesis [5] that has shown an unequal recovery of health-related quality of life after the acute phase [16]. Therefore, it is important to recognise those patients who are at risk of having long-lasting ED after COVID-19 recovery. The preservation of erectile function is critical because, apart from its sexual aspect, it is a marker of cardiovascular [12,17] and general health [17]. Thus, ED patients could be screened for cardiovascular diseases and eventually be treated in order to avoid the associated complications.

Despite knowing that ED following COVID-19 can persist even 12 months after recovery, no strategies have been designed to perform the early detection of this condition using recovered COVID-19 patients. Our work aimed to develop a risk predictive model for the identification of features involved in the prediction of ED 12 months after COVID-19 recovery. The main contribution of our research is the creation of a clinical tool to determine the key issues that influence most ED at 12 months following COVID-19 recovery.

## 2. Materials and Methods

### 2.1. Study Design

We carried out a prospective observational multicentre investigation in the following institutions: Clinic University Hospital of Valladolid, the Río Hortega University Hospital and the Santa Barbara Hospital. This study was approved by the ethics committees of all three institutions: CEIC Área de Salud Valladolid Este (cod: PI-GR-21-2405), CEIC Área de Salud Valladolid Oeste (cod: 21-PI211) and CEIC Área de Salud Burgos y Soria (cod: 2873). We performed the study in accordance with the ethical standards of the Declaration of Helsinki. Patients signed written informed consent prior to being enrolled in the study and were classified according to their history of COVID-19. For the cohort of patients with a past history of COVID-19, we enrolled men aged 40–70 years hospitalised for COVID-19 and discharged from hospital between 1 January 2021 and 31 March 2022. In these patients, hospital admission decisions were based on a COVID-19 diagnosis (established on RT-PCR from nasopharyngeal swab) in addition to clinical or radiological findings [fever resistant to medical treatment; pneumonia diagnosed by X-ray or CT] and followed the institutional protocols concerning COVID-19 management of each of the participant centres at the time of the study. For the cohort of patients without a past history of COVID-19, we enrolled men aged 40–70 years with neither a past history of microbiologic diagnosis of COVID-19 nor a past history of hospital admission in the last 12 months who were recruited in a urology outpatient consultation between 1 January 2022 and 31 March 2023. Convenience sampling was used due to the limited availability of cases of interest. Exclusion criteria were a past history of rectal, urethral, prostate or penile prosthesis surgery and a past history of ED. We declared those individuals who had not experienced sexual intercourse in the month before the assessment to be non-sexually active, and they were also excluded.

### 2.2. Outcomes

We assessed ED in both groups using the 5-item International Index of Erectile Function (IIEF-5) [18], which is a self-reporting instrument for the evaluation of male sexual function using five questions focused on erectile function. Designed for easy use by physicians in the clinical settings [18], it is a powerful tool as it allows the diagnosis of ED to be stablished [18], and it has been used in a wide variety of scientific works [19,20,21]. According to the standardised use of the instrument, ED is diagnosed when a score of 5–21 is obtained, while a score of 22–25 dismisses the diagnosis of ED [18]. We collected data concerning epidemiological variables (age, BMI, highest level of education, occupation, physical activity, civil status, living arrangements and SARS-CoV-2 vaccination), habits (smoking, alcohol, coffee and cannabis), comorbidities (ischemic heart disease, high blood pressure [hypertension], atrial fibrillation, heart failure, stroke, peripheral arterial disease [PAD], diabetes, obstructive sleep apnoea syndrome [OSAS], chronic obstructive pulmonary disease [COPD], asthma, hypothyroidism, chronic kidney failure, chronic active hepatitis, Parkinson’s disease, cancer, anxiety/depression, lower urinary tract symptoms [LUTS] and autoimmune disease) and active treatments (beta blockers, statins, non-thiazide diuretics, spironolactone, corticosteroids, antiplatelet therapy, anticoagulation therapy [acenocumarol or new oral anticoagulants], low-molecular-weight heparin [LMWH], benzodiazepines, antidepressants, antipsychotics, anticholinergics and alpha-1 receptor antagonists or 5-alpha reductase inhibitors). Intensive care unit (ICU) transfer data during hospitalisation for the worsening of symptoms were collected for the past history of COVID-19 cohort. We defined smokers as all patients who were active (regular cigarette smoking for a duration > 6 months) or former smokers (no history of smoking for >6 months) at the time of the assessment and quantified this through the use of the pack-year measure. Cannabis consumption was declared for patients that reported having smoked cannabis ≥ 3 times/week in the month before the assessment. Coffee consumption was defined on a daily intake of at least one cup of regular coffee/day at the time of assessment.

### 2.3. Data Collection

We designed a questionnaire with questions regarding the outcomes described previously. Patients completed the questionnaire through a telephone interview or filled in it using Google Forms (https://forms.gle/eog9K9g4mjT8CUJC8; https://forms.gle/ANZ2eREmiAtzdFS39; accessed on 26 September 2024) between 1 January 2022 and 31 March 2023.

### 2.4. Data Analysis

First, we performed a descriptive analysis of the previously described variables. Results were reported as medians (interquartile range) for continuous variables and as frequencies and percentages for categorical variables. Empty records or those filled with null data were eliminated.

#### 2.4.1. Test of Proportions

We calculated the prevalence of ED in each of the cohorts and performed a test of proportions in order to compare the differences in the proportions of ED for both cohorts. The null hypothesis stated that the proportions of positive cases of ED were equal in the two cohorts, but the alternative hypothesis stated that they were different. The level of confidence in our *z*-hypothesis test was 0.95 and the equation for the test of proportions (*z*-test) carried out was as follows:

(1) z_test= (x1/n1−x2/n2)/√(p(1−p)(1/n1+1/n2))

*p* = proportion of the sample
*x*1 = success in the no past history of COVID-19 cohort
*x*2 = success in the past history of COVID-19 cohort
*n*1 = observations in the no past history of COVID-19 cohort
*n*2 = observations in the past history of COVID-19 cohort

#### 2.4.2. Regression Model

**Figure 1:**
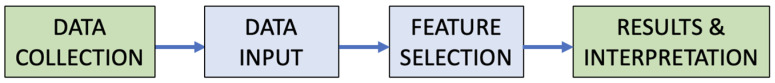
Research flow chart showing the procedures implemented for the creation of the model.

A logistic regression model for the selection of the variables involved in the prediction of ED was used in our sample; the following methodology was applied (Figure 1).

ED is a dichotomous variable, with 0 indicating a non-success and 1 denoting a success in a set of Bernoulli experiments following a binomial distribution. Therefore, we opted for a statistical model that fitted the intrinsic properties of this variable: a binary logistic regression model.

At first, we used Pearson’s correlation to eliminate highly correlated variables. The correlation coefficient is a number that expresses the degree of dependence of one set with respect to another. In mathematical terms, we can consider the Pearson’s correlation coefficient to be the normalised covariance; this is denoted by the Greek letter *ρ* (ro) as shown in the following equation:

(2) $$\rho xy=\frac{Cxy}{\sigma x\sigma y},$$

where Cxy is the covariance and σ are the respective standard deviations of the groups of variables. This value is in the interval [−1, 1] and can be interpreted as follows: When *ρ* = 1, the two sets maintain a linear dependence; when *ρ* = −1, the two sets maintain an inverse linear dependence and when *ρ* = 0, they are considered orthogonal signals. The coefficient threshold used for the dropout process in our study was 0.8.

Binary logistic regression is a statistical technique that aims to test hypotheses or causal relationships when the dependent variable, or the variable to be predicted, is a binary variable that has only two categories (success or failure). Although its reading resembles multiple linear regression, which is used when the dependent variable is ordinal or scalar, logistic regression is based on probabilities. In this technique, the independent variables attempt to predict the probability that something will occur over the probability that it will not occur. The logistic regression model can be described as shown in Equation (3):

(3) $$log\frac{p(X)}{1-p(X)}=w_{1}x_{1}+w_{2}x_{2}+w_{3}x_{3}\ldots \;w_{n}x_{n}$$

By subtracting the probability $p\left(x\right)$ from Equation (2), we obtain Equation (4):

(4) $$p\left(x\right)=\frac{e^{s}}{1+e^{s}};\;s=w_{1}x_{1}+w_{2}x_{2}+w_{3}x_{3}\ldots \;w_{n}x_{n}$$

By using this model, we can use an optimisation algorithm to minimise the error between the desired probabilities, adjusting the vector of weights W. Each of the variables is related to an associated weight ($w_{n}$), which determines its importance. These weights are the input for an RFE algorithm, which trains the model recursively until the desired number of variables is obtained. To measure the performance of the model obtained, the standard metrics of Area Under the Curve (AUC) and the Receiver Operator Characteristic (ROC) curve are used. We then performed a two-factor factorial design of experiments, as shown in Table 1, in order to collect the necessary data.

We used the programming language Python (from Python Software Foundation) version 3.8 and the Sklearn (Version 1.1) package libraries [22] to perform the descriptive analysis and the regression model.

## 3. Results

**Figure 2:**
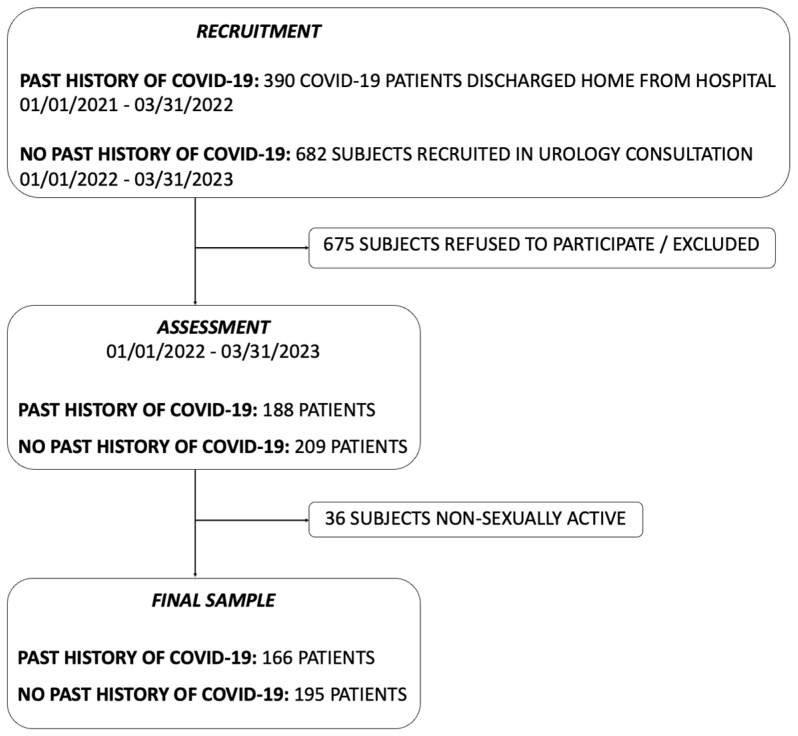
Flow chart showing the process for selection of the study sample. After the identification of the eligible individuals, we applied the exclusion criteria and then withdrew those individuals who were declared to be non-sexually active. We finally achieved the final study sample.

We identified 1072 eligible patients. Following the application of exclusion criteria and the exclusion of non-sexually active individuals, we obtained the final sample for our study (Figure 2).

### 3.1. Descriptive Analysis

The individuals with a past history of COVID-19 completed the questionnaire a median of 374 days (interquartile range 20) after discharge from hospital. The median age was 55 years in both cohorts. The descriptive analysis of the most relevant variables is shown in Table 2. The detailed descriptive analysis of all of the variables investigated is shown in Table S1 (Supplementary Materials).

### 3.2. Test of Proportions

The prevalence of ED was higher in the COVID-19 group (55.9%) than in the control group (44.1%). The test of proportions carried out (shown in Table 3) rejected the null hypothesis (*p* = 7.4 × 10^−5^).

### 3.3. Regression Model

Following the application of Pearson’s correlation test, we eliminated the following variables: non-thiazide diuretics (correlation coefficient = 0.9), benzodiazepines (correlation coefficient = 0.9) and antipsychotics (correlation coefficient = 0.8).

The best AUC was achieved for the model with 40 variables (AUC = 0.8). The top 15 variables in the model are shown in Table 4. A complete description of all variables of the model is shown in Table S2 (Supplementary Materials).

## 4. Discussion

To the best of our knowledge, this is the first time a predictive model has been developed for the prediction of ED at 12 months following COVID-19 recovery. A past history of COVID-19 was shown to be an independent predictor of ED in our sample.

Given that ED is detected in recovered COVID-19 patients, even 12 months after the disease, one question remains unsolved: Can we predict who will be affected by ED? Both COVID-19 and ED are complex diseases whose aetiology might be challenging to analyse. In our study, we opted for a binary logistic regression approach, which allowed us to create a predictive model for ED. The model developed showed adequate performance in our population, with an AUC of 0.8. The use of predictive models in COVID-19 recovered patients could help to identify men who might be at risk of developing ED. The mechanism of post-COVID-19 ED might be multifactorial, as described by Zhang [14], with the impairment of the endothelial function being one of the described causes [23,24]. We know that ED might be the first symptom of a silent cardiovascular disease [17,21], and it has been proposed that post-COVID-19 ED patients should undergo a comprehensive cardiovascular assessment [14]. In this way, the implementation of our predictive model could allow the early detection of these individuals.

A past history of COVID-19 behaved as an independent predictor of ED (weight $e^{w_{i}}=$ 1.3). This is a pivotal finding because it demonstrates the relationship between COVID-19 and ED, even 12 months after the disease. The selection of a past history of COVID-19, among the features of our predictive model, which is actually one of the most important, reveals the long-lasting impact and influence of COVID-19 on general health. As such, our findings would take COVID-19 to the level of other well-known determinants of ED [25]. SARS-CoV-2 vaccination was also one of the features selected in our model. Although a link between SARS-CoV-2 vaccination and long COVID has been described [26], there are few works that have specifically investigated the link between SARS-CoV-2 vaccination and ED to date. Mehta et al. reported that the SARS-CoV-2 vaccination had no adverse effect on erectile function [27], but the sample did not include recovered COVID-19 patients. Given the known adverse effects of COVID-19 on ED, it would be interesting to assess how SARS-CoV-2 vaccination interacts with erectile function in recovered patients, who might already have some level of ED. Diaz et al. concluded that erectile function between vaccinated and unvaccinated individuals was not statistically different [28]. If we look into their work, although recovered COVID-19 patients were enrolled, no specific analysis regarding their relationship with SARS-CoV-2 vaccination and ED was described [28]. Our findings regarding SARS-CoV-2 vaccination and the prediction of ED are purely clinical and not supported by molecular methods. We could not collect information about the number of vaccination doses or the time from COVID-19 recovery to vaccination, which are interesting facts. Therefore, they should be interpreted cautiously.

We found that the prevalence of ED was higher in the past history of COVID-19 cohort (55.9% vs. 44.1%), while the proportions test carried out demonstrated statistical significance (*p* = 7.4 × 10^−5^). A relationship between COVID-19 and ED was described for the first time in 2020 [12] and later in other works [13,14]. Moreover, Gök et al. reported an impairment of erectile function outcomes even 12 months after COVID-19 recovery [15]. Our findings support the results obtained by these authors. We were not able to collect baseline erectile function data prior to COVID-19 due to the design of our study, but we selected a cohort of patients with no past history of COVID-19 in order to balance this. The ED rate in that cohort (44.1%) was similar to the described Spanish ED prevalence rates (43.5%) [1]. In addition, the individuals with previously diagnosed ED were excluded from the study.

Erectile function is a marker of general health [12,17], while ED is considered a strong predictor of cardiovascular events [21,29]. Prior to 3 October 2023, there have been more than 600,000,000 known COVID-19 cases worldwide [30]. Given the high global COVID-19 incidence and the unlikely eradication of the disease in the future [31,32], more complications and sequels are expected in the coming months and years. Our predictive model could be decisive in the prediction of ED following COVID-19 in community settings. The application of our predictive tool in other recovered COVID-19 patients would lead to the prompt identification of individuals at risk of developing ED. They could be then subjected to a comprehensive cardiovascular assessment, which could avoid the onset of cardiovascular diseases and, consequently, the adverse impact on healthcare systems.

Some limitations must be described. The data of interest were collected through a survey, trusting the facts described by participants. Our predictive model should be externally validated in other populations. Future investigations should include specific analysis about the effect of SARS-CoV-2 vaccination on the erectile function of recovered COVID-19 patients, a statistical analysis to evaluate the influence of the comorbidities of COVID-19 recovered patients on ED and sharing datasets about ED in recovered COVID-19 patients. This would allow the creation of large multicentric databases, leading to the application of predictive tools involved in ED screening. A reduction in the utilisation of health resources and the socioeconomic impact of the disease could then be seen.

## 5. Conclusions

In conclusion, we developed a regression model for the prediction of ED at 12 months following COVID-19 recovery. The application of our predictive tool in recovered COVID-19 patients in a community setting could eventually lead to avoidance of the adverse effects of ED and the associated unfavourable economic impact. A future line of investigation should include the specific assessment of the effect of SARS-CoV-2 vaccination and other comorbidities on the erectile function of recovered COVID-19 patients.

## Figures and Tables

**Table 1:** Two-factor factorial experiment designed to measure the performance of the predictive models obtained. The factor model had 8 levels, since the number of attributes that the algorithm chose as the most important to predict the selected variable ranged from 5 to 40. For each of the levels, we calculated the AUC metrics and the respective ROC curve was generated. The factor variable to predict had one level: ED.

Factor 1/Factor 2		Variable to Predict
		Ed
Model	Logistic Regression for Selection of 5 Attributes	AUC, ROC
	…	…
	Logistic Regression for Selection of 40 Attributes	AUC, ROC

ED: Erectile dysfunction; AUC: Area Under the Curve; ROC: Receiver Operator Characteristics.

**Table 2:** Descriptive analysis of the most important characteristics of the patients enrolled in the study.

Variable	History of COVID-19	
	No (*n* = 195)	Yes (*n* = 166)
Age (year, interquartile range)	55 (15)	55 (14)
BMI (kg/m^2^, interquartile range)	26.3 (5)	28 (6)
Smoking (case, %)	94 (48.5)	100 (51.5)
Pack-year (pack year, interquartile range)	18 (14)	25.5 (25)
Ischemic heart disease (case, %)	5 (25)	15 (75)
Hypertension (case, %)	45 (44.1)	57 (55.9)
Heart failure (case, %)	1 (16.7)	5 (83.3)
PAD (case, %)	4 (50)	4 (50)
Diabetes (case, %)	10 (33.3)	20 (66.7)
Hypothyroidism (case, %)	6 (60)	4 (40)
CKD (case, %)	6 (75)	2 (25)
Cancer (case, %)	6 (40)	9 (60)
Anxiety/Depression (case, %)	6 (23.1)	20 (76.9)
Beta blockers (case, %)	6 (18.8)	26 (81.3)
NOAs (case, %)	1 (16.7)	5 (83.3)
Antiplatelet therapy (case, %)	12 (37.5)	20 (62.5)
Erectile dysfunction * (case, %)	83 (44.1)	105 (55.9)
SARS-CoV-2 vaccination (case, %)	191 (54.6)	159 (45.4)

Values are expressed as number (%) and median (interquartile range). BMI = body mass index; PAD = peripheral arterial disease; CKD = chronic kidney failure; NOAs = new oral anticoagulants. * Erectile dysfunction missing data on four patients.

**Table 3:** The test of proportions carried out showed statistical significance and, therefore, rejected the null hypothesis.

Statistic	Values
No past history of COVID-19	83
Past history of COVID-19	105
N No past history of COVID-19	193
N past history of COVID-19 cohort	164
P No past history of COVID-19 %	43
P Past history of COVID-19 %	64
N	357
*p*-value	7.4 × 10^−5^
z-value	−3.9640

**Table 4:** Among the 15 variables with the highest weight in the ED predictive model, which are shown in the table, a past history of COVID-19 was selected with a weight ($e^{w_{i}}$) of 1.3.

Erectile Dysfunction Predictive Model		
Index	Variable	$\mathrm{Weight}\;e^{w_{i}}$
1	Diabetes	3.7
2	Autoimmune disease	2.5
3	Beta blockers	2.3
4	PAD	1.8
5	Cancer	1.8
6	Hypertension	1.8
7	Stroke	1.7
8	Chronic kidney failure	1.7
9	Acenocumarol	1.7
10	Antiplatelet therapy	1.5
11	Anxiety/Depression	1.5
12	COPD	1.4
13	Alcohol	1.3
14	History of COVID-19	1.3
15	Hypothyroidism	1.3

PAD: peripheral arterial disease; COPD: chronic obstructive pulmonary disease.

## Data Availability

The raw data supporting the conclusions of this article will be made available by the authors on request.

## Supplementary Materials

The following supporting information can be downloaded at: https://www.mdpi.com/article/10.3390/jcm13195757/s1, Table S1: Descriptive analysis of the characteristics of the patients enrolled in the study; Table S2: Variables included in the predictive model for ED.
