# Supplementary material for: Development of a Risk Predictive Model for Erectile Dysfunction at 12 Months after COVID-19 Recovery: A Prospective Observational Study
Source: J Clin Med. 2024 Sep 27;13(19):5757. doi: 10.3390/jcm13195757 (PMC11477077; doi:10.3390/jcm13195757)
Supplement: Supplementary file 1 [file jcm-13-05757-s001.zip › Supplementary material Table S1.pdf]

Table S1. Characteristics of the patients of the final sample.

VARIABLE		HISTORY OF COVID-19	
		NO (*N*= 195)	YES (*N*=166)
Age (year, interquartile range)		55 (15)	55 (14)
BMI (kg/m^2)		26.3 (5)	28 (6)
Highest level of education (case (%))	Primary	25 (34.7)	47 (65.3)
	Secondary	43 (37.4)	72 (62.6)
	University	126 (73.3)	46 (26.7)
Occupation (case (%))	Directors	8 (34.8)	15 (65.2)
	Scientists	100 (73)	37 (27)
	Technicals	6 (37.5)	10 (62.5)
	Accountants	10 (62.5)	6 (37.5)
	Restauration	16 (51.6)	15 (48.4)
	Agriculture	1 (12.5)	7 (87.5)
	Industry	20 (44.4)	25 (55.6)
	Assemblers	3 (17.6)	14 (82.4)
	Elementary	1 (25)	3 (75)
	Military	2 (100)	0 (0)
	Retirement	25 (44.6)	31 (55.4)
	Unemployed	3 (50)	3 (50)
Physical activity (case (%))	Low	96 (50.8)	93 (49.2)
	Moderate	78 (55.3)	63 (44.7)
	High	21 (67.7)	10 (32.3)
Civil status in couple (case (%))		173 (55.3)	140 (44.7)
Living arrangements (case (%))	Unipersonal	17 (45.9)	20 (54.1)
	Monoparental	4 (66.7)	2 (33.3)
	Couple without children	79 (55.2)	64 (44.8)
	Couple with children	9 (42.9)	12 (57.1)
Alcohol (case (%))		97 (67.8)	46 (32.2)
Smoking (case (%))		94 (48.5)	100 (51.5)
Coffee (case (%))		159 (59.1)	110 (40.9)

Ischemic heart disease (case (%))	5 (25)	15 (75)
Hypertension (case (%))	45 (44.1)	57 (55.9)
Atrial fibrillation (case (%))	6 (35.3)	11 (64.7)
Heart failure (case (%))	1 (16.7)	5 (83.3)
Stroke (case (%))	4 (30.8)	9 (69.2)
PAD (case (%))	4 (50)	4 (50)
Diabetes (case (%))	10 (33.3)	20 (66.7)
OSAS (case (%))	11 (57.9)	8 (42.1)
COPD (case (%))	3 (33.3)	6 (66.7)
Hypothyroidism (case (%))	6 (60)	4 (40)
Asthma (case (%))	12 (63.2)	7 (36.8)
Chronic kidney failure (case (%))	6 (75)	2 (25)
Chronic active hepatitis (case (%))	4 (66.7)	2 (33.3)
Parkinson disease (case (%))	0 (0)	0 (0)
Cancer (case (%))	6 (40)	9 (60)
Anxiety/Depression (case (%))	6 (23.1)	20 (76.9)
LUTS (case (%))	22 (56.4)	17 (43.6)
Autoimmune disease (case (%))	2 (25)	6 (75)
Statins (case (%))	29 (42)	40 (58)
Non-thiazide diuretics (case (%))	39 (46.4)	45 (53.6)
Beta blockers (case (%))	6 (18.8)	26 (81.3)
Spironolactone (case (%))	1 (50)	1 (50)
Corticoids (case (%))	2 (66.7)	1 (33.3)
Acenocumarol (case (%))	5 (50)	5 (50)
New oral anticoagulants (case (%))	1 (16.7)	5 (83.3)
LMWH (case (%))	0 (0)	0 (0)
Antiplatelet therapy (case (%))	12 (37.5)	20 (62.5)
Benzodiazepines (case (%))	6 (26.1)	17 (73.9)
Antidepressants (case (%))	1 (16.7)	5 (83.3)
Anticholinergics (case (%))	5 (62.5)	3 (37.5)
A1RA/5-ARI (case (%))	21 (55.3)	17 (44.7)
Antipsychotics (case (%))	3 (100)	0 (0)
ICU admission (case (%))	-	24 (14.5)
ED (case (%))	83 (44.1)	105 (55.9)

SARS-cov-2 Vaccination	191 (54.6)	159 (45.4)

Values are expressed as number (%) and median (interquartile range). BMI=body mass index; PAD=peripheral arterial disease. OSAS=obstructive sleep apnoea syndrome. COPD=chronic obstructive pulmonary disease. LMWH=low molecular weight heparin. A1RA=alfa-1 receptors antagonists. 5-ARI=5-alfa reductase inhibitors. ICU=intensive care unit. ED=Erectile dysfunction.
