# Supplementary material for: Development of a Risk Predictive Model for Erectile Dysfunction at 12 Months after COVID-19 Recovery: A Prospective Observational Study
Source: J Clin Med. 2024 Sep 27;13(19):5757. doi: 10.3390/jcm13195757 (PMC11477077; doi:10.3390/jcm13195757)
Supplement: Supplementary file 1 [file jcm-13-05757-s001.zip › Supplementary material Table S2.pdf]

Table S2. Variables included in the predictive model for ED.

ERECTILE DYSFUNCTION PREDICTIVE MODEL		
Index	Variable	Weight e^{w_i}
1	Diabetes	3.7
2	Autoimmune disease	2.5
3	Beta blockers	2.3
4	PAD	1.8
5	Cancer	1.8
6	Stroke	1.7
7	Hypertension	1.8
8	Chronic kidney failure	1.7
9	Acenocumarol	1.7
10	Antiplatelet Therapy	1.5
11	Anxiety / depression	1.5
12	COPD	1.4
13	Alcohol	1.3
14	History of COVID-19	1.3
15	Hypothyroidism	1.3
16	Chronic active hepatitis	1.2
17	Physical activity	1.2
18	Smoking	1.2
19	Heart failure	1.1
20	Atrial fibrillation	1.1
21	Age	1.1
22	Anticholinergics	1.1
23	Occupation	1
24	ICU admission	1
25	Spironolactone	1
26	Corticoids	1
27	BMI	1
28	LUTS	1
29	Statins	1
30	Highest level of education	0.8
31	SARS-CoV-2 vaccination	0.8
32	Asthma	0.8
33	New oral anticoagulants	0.8
34	OSAS	0.7
35	Cannabis	0.7
36	Living arrangements	0.7
37	Antidepressants	0.7
38	Ischemic heart disease	0.7
39	Coffee	0.6
40	Civil status in couple	0.6

PAD=peripheral arterial disease. COPD=chronic obstructive pulmonary disease. ICU=intensive care unit. BMI=body mass index. OSAS=obstructive sleep apnoea syndrome.
